# Missing Opportunity for Nephroprotective Therapy in Patients With Non-Dialysis CKD Under Stable Nephrology Care

**DOI:** 10.1016/j.ekir.2026.106541

**Published:** 2026-04-14

**Authors:** Luca De Nicola, Chiara Ruotolo, Gaetano La Manna, Vincenzo Bellizzi, Loreto Gesualdo, Felice Nappi, Pietro Manuel Ferraro, Carmelita Marcantoni, Domenico Santoro, Giovanni Stallone, Antonio Pisani, Cataldo Abaterusso, Adamasco Cupisti, Luigi Vernaglione, Maura Ravera, Mario Cozzolino, Maurizio Gallieni, Federico Alberici, Marcellino Corvinelli, Yuri Battaglia, Alessandro Capitanini, Filippo Aucella, Giuseppe Castellano, Mariadelina Simeoni, Michele Provenzano, Mario Bonomini, Gianpaolo Reboldi, Angelo Ferrantelli, Ciro Esposito, Gianfranca Cabiddu, Giuseppe Grandaliano, Roberto Minutolo, REport of Nondialysis patients followed-up with in Italian nephrology clinics to Enable Winning strategy in CKD (RENEW-CKD) study group of Italian Society of Nephrology, Loreto Gesualdo, Pietro Cirillo, Alessandro Mascolo, Silvia Porreca, Giuseppe Scarimbolo, Davide Morisco, Simone Di Pace, Gaetano La Manna, Irene Capelli, Miriam Di Nunzio, Federico Alberici, Federica Mescia, Francesco Ravelli, Martina Tedesco, Marco Gregori, Luigi Vernaglione, Flavia Capaccio, Maria Teresa Farina, Domenica Biasi, Giuseppe Leonardi, Mariagrazia Arcidiacono, Lucia Argentiero, Alessandra Spinelli, Gianfranca Cabiddu, Stefania Maxia, Stefania Caira, Marcellino Corvinelli, Lucio Polese, Vincenzo Bellizzi, Caterina Saviano, Ida Molfino, Pasqualina Acconcia, Carmelita Marcantoni, Simone Di Lorenzo, Irene Torrisi, Roberta Aliotta, Grazia Portale, Mario Bonomini, Bianca Lo Giudice, Emanuele Marini, Lorenzo Nolletti, Valeria Vezzani, Vittorio Sirolli, Michele Provenzano, Chiara Summaria, Fiorella Iorio, Francesco Zingone, Giuseppe Pezzi, Roberta Arena, Giovanni Stallone, Dario Troise Barbara Infante, Maura Ravera, Domenico Santoro, Chiara Casuscelli, Michela Calderone, Felicia Cuzzola, Maurizio Gallieni, Luca Della Volpe, Michela Tedesco, Alessia Guarino, Laura Cosmai, Marco Heidempergher, Sabrina Caruso, Mario Cozzolino, Sara Masotto, Anna Maria Ferrigno, Clorinda Rossi, Chiara Cuffaro, Giuseppe Castellano, Simone Vettoretti, Luca De Nicola, Roberto Minutolo, Paolo Tino Ambrosino, Simona Andriella, Davide Cesarano, Annarita D’Ambra, Federica Marzano, Christian Nardelli, Chiara Ruotolo, Antonio Pisani, Fabio Esposito, Michela Saggese, Giuditta della Corte, Mariadelina Simeoni, Davide Loffredo, Angelo Savino, Felice Nappi, Carla Clienti, Antonietta La Verde, Angelo Ferrantelli, Ciro Esposito, Marta Arazzi, Vittoria Esposito, Filippo Sangregorio, Gianpaolo Reboldi, Sara Battistoni, Nicola Lommano, Manuel Burdese, Yuri Battaglia, Federica Baciga, Michela Erlati, Veronica Brunelli, Adamasco Cupisti, Domenico Giannese, Claudia D’Alessandro, Vincenzo Panichi, Alessandro Capitanini, Adriano Carmelo Piluso, Dritan Curi, Elana Romoli, Vincenzo Miniello, Brunilda Xhaferi, Giuseppe Grandaliano, Viola D’Ambrosio, Filippo Aucella, Rachele Grifa, Maria Nardella, Pérez Ys Aurora Del Mar, Cataldo Abaterusso, Michelangelo Beltrami, Marta Guizzo, Roberta Lazzarin, Mariapaola Protti, Claudia Ungaro, Pietro Manuel Ferraro, Isabella Squarzoni, Andrea Spasiano, Francesco Bonetti

**Affiliations:** 1 Division of Nephrology, Department of Advanced Medical and Surgical Sciences, University of Campania “Luigi Vanvitelli,” Naples, Italy; 2 Nephrology, Dialysis and Renal Transplant Unit, IRCCS Azienda Ospedaliero-Universitaria di Bologna, Bologna, Italy; 3 Department of Medical and Surgical Sciences, Alma Mater Studiorum University of Bologna, Bologna, Italy; 4 Division of Nephrology "Sant'Anna e San Sebastiano" Hospital, Caserta, Italy; 5 Department of Precision and Regenerative Medicine and Ionian Area, Department Emergency and Organ Transplantation, University of Bari Aldo Moro, Bari, Italy; 6 Nephrology and Dialysis Unit, S. Maria della Pietà Hospital, Nola, Italy; 7 Section of Nephrology, Department of Medicine, University of Verona, Verona, Italy; 8 Nephrology Unit, Azienda Ospedaliera Universitaria Integrata Verona, Verona, Italy; 9 Nephrology and Dialysis Unit, Azienda Ospedaliero-Universitaria “G. Rodolico-San Marco” di Catania, Catania, Italy; 10 Nephrology and Dialysis Division, University of Messina, Messina, Italy; 11 Nephrology, Dialysis, and Transplantation Unit, Department of Medical and Surgical Sciences, University of Foggia, Foggia, Italy; 12 Nephrology Unit, Department of Public Health, University Federico II of Naples, Naples, Italy; 13 Nephrology and Dialysis Unit, Civil Hospital of Castelfranco Veneto, Treviso, Italy; 14 Department of Clinical and Experimental Medicine, University of Pisa, Pisa, Italy; 15 Nephrology and Dialysis Unit, "A. Perrino" Hospital of Brindisi, Brindisi, Italy; 16 Nephrology, Dialysis, and Transplantation, Policlinico San Martino, Genoa, Italy; 17 Renal Division, Department of Health Sciences, University of Milan, ASST Santi Paolo e Carlo, Milan, Italy; 18 Nephrology and Dialysis Unit, Department of Biomedical and Clinical Sciences, University of Milano, ASST Fatebenefratelli Sacco, Milan, Italy; 19 Nephrology Unit, University of Brescia-ASST Spedali Civili di Brescia, Brescia, Italy; 20 Nephrology and Dialysis Unit, Hospital “A. Cardarelli,” Campobasso, Italy; 21 Nephrology and Dialysis Unit, Pederzoli Hospital, Peschiera del Garda, Italy; 22 Nephrology and Dialysis Unit, ASL Toscana Centro, Pistoia, Italy; 23 Nephrology and Dialysis Unit, IRCCS Ospedale Casa Sollievo della Sofferenza, San Giovanni Rotondo, Italy; 24 IRCCS Ca' Granda Ospedale Maggiore Policlinico, Milan, Italy; 25 Department of Translational Medical Sciences, University of Campania “Luigi Vanvitelli,” Naples, Italy; 26 Department of Pharmacy, Health, and Nutritional Sciences, University of Calabria, Rende, Cosenza, Italy; 27 Department of Medicine, G. d'Annunzio University of Chieti-Pescara, Chieti, Italy; 28 Department of Medicine and Surgery, University of Perugia, Perugia, Italy; 29 Nephrology, Dialysis, and Kidney Transplant Unit, ARNAS Ospedale Civico Palermo, Palrmo, Italy; 30 Unit of Nephrology, Istituti Clinici Scientifici Maugeri IRCCS, Pavia, Italy; 31 University of Pavia, Pavia, Italy; 32 Department of Medical Sciences and Public Health, University of Cagliari, Cagliari, Italy; 33 Nephrology Dialysis and Renal Transplantation, IRCCS "A. Gemelli" University Hospital, Rome, Italy

**Keywords:** albuminuria, chronic kidney disease, hypertension, risk factors, therapeutic inertia

## Abstract

**Introduction:**

Awareness of the implementation of guideline-directed nephroprotective therapy is an essential preliminary step to optimize implementation of nephroprotective strategies in nondialysis chronic kidney disease (CKD) (ND-CKD). However, updated information on this issue is lacking in the setting of nephrology clinics.

**Methods:**

In this multicenter prospective study, we collected data from 4523 patients with ND-CKD, either stage 3 to 5 or 1 and 2 with urinary albumin-to-creatinine ratio (ACR) > 30 mg/g, followed up in 30 Italian nephrology clinics. Patients were evaluated at 2 visits with a 6-month interval between May 2024 and May 2025. The aim was to evaluate the current phenotype of patients under tertiary nephrology care and the management of the 2 major modifiable determinants of renal risk, hypertension and ACR, including therapeutic inertia.

**Results:**

The cohort was characterized by a severe cardiorenal risk profile: men comprised 65% of the cohort, mean age was 71 ± 14 years, diabetes was present in 40%, cardiovascular disease in 40%, estimated glomerular filtration rate (eGFR) was 34 ± 19 ml/min per 1.73 m^2^, and ACR was 70 mg/g (interquartile range: 11–350). At the 6-month visit, in patients with and without diabetes, home and office blood pressure (BP) were above target in about 70% of patients, with a high prevalence of sustained (62%) and resistant (23%) hypertension. Among patients with uncontrolled office BP, 33% and 43% of patients with and without diabetes, respectively, were prescribed ≤ 2 BP-lowering drugs. ACR > 30 mg/g persisted in 61% of nondiabetic and 64% of patients with diabetes. Therapeutic inertia for antialbuminuric agents at month 6 was frequent: 85% for renin-angiotensin system (RAS) inhibitors and 90% for gliflozins. Among patients with diabetes, therapeutic inertia was 92% for glucagon-like peptide-1 receptor agonists (GLP1-RAs) and 96% for finerenone.

**Conclusion:**

The large majority of patients with ND-CKD currently followed up in Italian renal clinics are characterized by a severe risk profile that is paradoxically associated with remarkable therapeutic inertia for both traditional and innovative guideline-directed nephroprotective therapy.

## Graphical abstract

**Figure:**
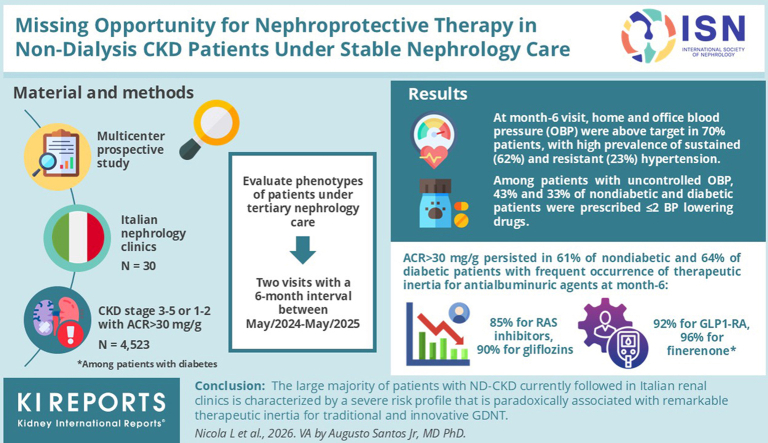

In the past 3 decades, nephrology has experienced a major paradigm shift in the vision of ND-CKD. The old view considered dietary and pharmacological intervention to lose most of their nephroprotective efficacy when serum creatinine reached levels as low as 1.5 to 2.0 mg/dl.^1^ This point-of-no-return idea was so pervasive that many nephrologists assimilated conservative treatment aimed at slowing the progression of CKD to palliative therapy prescribed to patients who cannot be treated or refuse dialysis. Conversely, nephrologists are now called to embrace a change in their practice, that is, preserving rather than substituting kidney function in order to postpone, as long as possible, the need for kidney replacement therapy (KRT).^2^

The main drivers for this epochal change are 2: first, the relentless growth of the KRT population, burdened by high morbidity and mortality as well as catastrophic costs, has made the prevention of KRT the primary task of modern nephrology^3^, ^4^, ^5^; second, solid proof supporting the efficacy of novel nephroprotective agents has emerged from several trials over the past decade.^6^ Accordingly, clinical practice guidelines, and in particular the widely disseminated guidelines on CKD management, the Kidney Disease: Improving Global Outcomes guidelines,^7^, ^8^, ^9^, ^10^, ^11^ have repeatedly issued recommendations on the need to implement these novel drugs to abate the risk of KRT. To meet this elevated target, identifying the areas of improvement in therapies aimed at preserving kidney function is mandatory. This issue becomes even more critical when considering that higher mortality is the “bad companion” of progressive CKD.^12^^,^^13^ Under this view, a key preliminary step is the knowledge of risk stratification and the degree of implementation of guideline-directed nephroprotective therapy.^6^ Although the best setting to investigate this issue is tertiary nephrology care, where patients with progressive CKD are referred, updated information on this critical issue is not available. Indeed, poor adherence to current guideline recommendations for delaying CKD progression has been mainly demonstrated in nonnephrology settings,^14^, ^15^, ^16^, ^17^, ^18^ whereas the studies carried out so far in nephrology clinics are outdated in terms of patient populations and the versions of the guidelines in use at the time.^19^, ^20^, ^21^, ^22^

To fill this important knowledge gap, we designed a multicenter prospective study enrolling a large contemporary cohort of patients with ND-CKD regularly followed up in outpatient nephrology clinics. The aim of this study was to evaluate the present phenotype of patients under tertiary nephrology care and the current management of the 2 major modifiable determinants of renal risk, hypertension, and albuminuria, in terms of drug prescription and therapeutic goal achievement.

## Methods

### Study Design

In 2024, the Italian Society of Nephrology sponsored the multicenter prospective study REport of Nondialysis patients followed in Italian nephrology clinics to Enable Winning strategy in CKD (RENEW-CKD) by recruiting 30 outpatient nephrology clinics distributed across Italy. The inclusion criteria for centers were as follows: the presence of an outpatient clinic dedicated to the conservative care of CKD, with an attending patient population seen at least twice/yr; and the availability of clinical and laboratory protocols for the management of these patients. According to the Italian Health Examination Survey on CKD, 155,000 patients with stage 3 to 5 CKD are referred to nephrologists^23^; therefore, we planned to enroll from Italian renal clinics, a sample equal to 3% of this estimated population (*n* = 4650 patients).

As verified in *ad hoc* meetings held before starting the data collection, all participating nephrologists agreed to follow the indications of the contemporary Kidney Disease: Improving Global Outcomes guidelines on ND-CKD management,^8^, ^9^, ^10^, ^11^ including interventions on nutritional approaches (salt intake < 6 g/d in hypertensive patients with CKD and protein intake ≤ 0.8 g/kg/d in patients with CKD stage 3–5). Regarding BP, centers agreed on the standardized protocol, including 2 physician-performed BP measurements taken 5 minutes apart in the sitting position after 10 minutes of rest using a cuff-oscillometric device, with Korotkoff phases I and V defining systolic and diastolic BP values, respectively. Office BP targets adopted in participating clinics were as follows: < 130/80 mm Hg in 27 of 30 centers (including 4376 patients), < 135/80 mm Hg (2 centers with 78 patients), and systolic BP < 130 mm Hg (1 center with 69 patients). Centers were also asked to register BP measurements attained at home when available. In this subgroup of patients, white-coat uncontrolled hypertension was diagnosed when office BP was high (≥130/80 mm Hg) and home BP was controlled (< 130/80 mm Hg); masked uncontrolled hypertension was defined as controlled hypertension in office (< 130/80 mm Hg) and high BP at home (≥ 130/80 mm Hg); and sustained hypertension was defined as high BP both in the office and at home, as recommended by the 2023 European Society of Hypertension Guidelines for the management of arterial hypertension.^24^

Each center enrolled consecutive patients with CKD stage 3 to 5 or stage 1 and 2 with ACR > 30 m/g who were referred to the clinics during a 6-month period between May 2024 and November 2024. Because the aim of the study was to evaluate patients steadily followed by nephrologists, we included only patients whose first visit to the renal clinic had occurred at least 6 months earlier. Exclusion criteria were KRT, acute kidney injury within the 6 months before baseline, life expectancy < 6 months (active malignancy, advanced liver or heart disease, or severe infectious disease), and missing information on proteinuria or albuminuria or eGFR. The study was approved by the ethical committee of the University of Campania Luigi Vanvitelli, and all patients signed informed consent before data collection.

### Procedures

For each patient, participating nephrologists collected demographic and medical history data, including diagnosis of diabetes mellitus (DM) and history of cardiovascular events, defined as the presence of coronary artery disease, congestive heart failure, cerebrovascular disease, or peripheral vascular disease. The diagnosis of underlying nephropathy was also requested; in patients with diabetes without kidney biopsy, DM was considered as the cause of CKD when, after exclusion of known nondiabetic kidney disease, both albuminuria and diabetic retinopathy were reported in clinical history. Clinical data, including therapy, and laboratory data were collected at the time of 2 visits, baseline and month 6, scheduled between May 2024 and November 2024 and December 2024 and May 2025, respectively, with the aim of exploring therapeutic inertia. Case report forms were completed at each center by participating nephrologists, keeping the patients’ identity anonymous, and sent to the coordinating center at the Nephrology Unit, University of Campania Luigi Vanvitelli, Naples, for quality control and data storage.

Laboratory protocols were standardized using in-house analyses. Twenty-four-hour urine collections were considered inaccurate and repeated if the value of measured creatinine excretion rate was outside the 60%–140% range of the value calculated according to Dwyer and Kenler.^25^ Daily salt intake (g/d) was calculated dividing 24-hour urinary sodium excretion as mmol by 17. eGFR was calculated using the 2009 CKD: Epidemiology Collaboration creatinine equation; in the case creatinine determination was not standardized to isotope-dilution mass spectrometry values, the serum levels were reduced by 5% according to Skali *et al.*^26^ Albuminuria was measured and calculated as ACR. If only proteinuria was available, the values obtained in either spot or 24-hour urine collections were extrapolated to ACR using a validated multivariable conversion equation.^27^ The evaluation of either proteinuria or albuminuria was not prespecified, and it was done according to the practice in each center.

### Definition of Risk Factors

At the time of the study visits, we assessed the prevalence of the 2 major modifiable renal risk factors, hypertension and albuminuria, as well as their related therapies. According to the 2023 European Society of Hypertension guidelines, uncontrolled hypertension was defined as office BP or home BP ≥ 130/80 mm Hg.^24^ Resistant hypertension (RH) was defined as office and home BP ≥ 130/80 mm Hg despite the prescription of ≥ 3 different classes of antihypertensive drugs (including diuretics) or BP controlled by means of ≥ 4 drugs.^28^ According to the Kidney Disease: Improving Global Outcomes,^8^, ^9^, ^10^, ^11^ albuminuria was classified as low (A1), moderate (A2), or severe (A3), defined as ACR < 30, 30–300, or > 300 mg/g, respectively; these cut-offs were adopted in all centers.

Therapeutic inertia at the month-6 visit for RAS inhibitors, sodium-glucose cotransporter 2 inhibitors (SGLT2i), GLP1-RAs, and finerenone was defined as failure to initiate therapy with each class despite the presence of an indication. It was assessed in patients not receiving each drug class at baseline but eligible to start treatment (either RAS inhibitors, SGLT2i, GLP1-RAs, or finerenone, individually evaluated) according to the reimbursement criteria of each drug at the time of data collection. In particular, eligibility for RAS inhibitors were defined as eGFR > 20 ml/min per 1.73 m^2^, ACR > 30 mg/g, and serum potassium < 5.0 mEq/l. Eligibility for SGLT2i were defined as eGFR of 20 to 60 ml/min per 1.73 m^2^, eGFR > 60 ml/min per 1.73 m^2^ with ACR > 30 mg/g, with no autosomal dominant polycystic kidney disease, no type 1 DM, and no active immunosuppressive treatment. Eligibility for GLP1-RAs was defined as type 2 DM with glycated hemoglobin > 7%. Eligibility for finerenone was defined as type 2 DM, eGFR of 25 to 60 ml/min per 1.73 m^2^, ACR > 30 mg/g, and serum potassium < 5.0 mEq/l. The prevalence of therapeutic inertia for each drug class was calculated as follows: (1 – [drug added at month 6/eligible patients] × 100). Among patients with DM, the assessment of therapeutic inertia was restricted to those with type 2 DM because SGLT2i, GLP1-RAs, and finerenone are not recommended in type 1 DM.

### Statistical Analysis

Continuous variables were reported as mean ± SD and compared using either paired or unpaired *t* test. Variables with a nonnormal distribution are reported as median (interquartile range) and analyzed using the Wilcoxon or Mann-Whitney test. Categorical variables are expressed as percentage and analyzed using either the McNemar test (paired data) or the chi-square test (unpaired data). Missing data for body mass index (0.7%), smoking habit (0.8%), home BP (65%), and urinary sodium excretion (79%) were not replaced.

Multivariable logistic regression analysis was used to identify factors associated with the presence of therapeutic inertia in the prescription of the 2 first-choice nephroprotective agents in patients with and without DM; that is, RAS inhibitors and SGLT2i, by including the following variables *a priori*: age, sex, body mass index, office systolic BP, DM, history of cardiovascular disease, ACR categories (A1–A3), and eGFR categories (> 60, 45–59, 30–44, and 20–30 ml/min per 1.73 m^2^).

A 2-tailed *P* value < 0.05 was considered significant. Data were analyzed using IBM SPSS Statistics for Windows, version 26.0 (IBM Corp, Armonk, NY).

## Results

**Figure 1:**
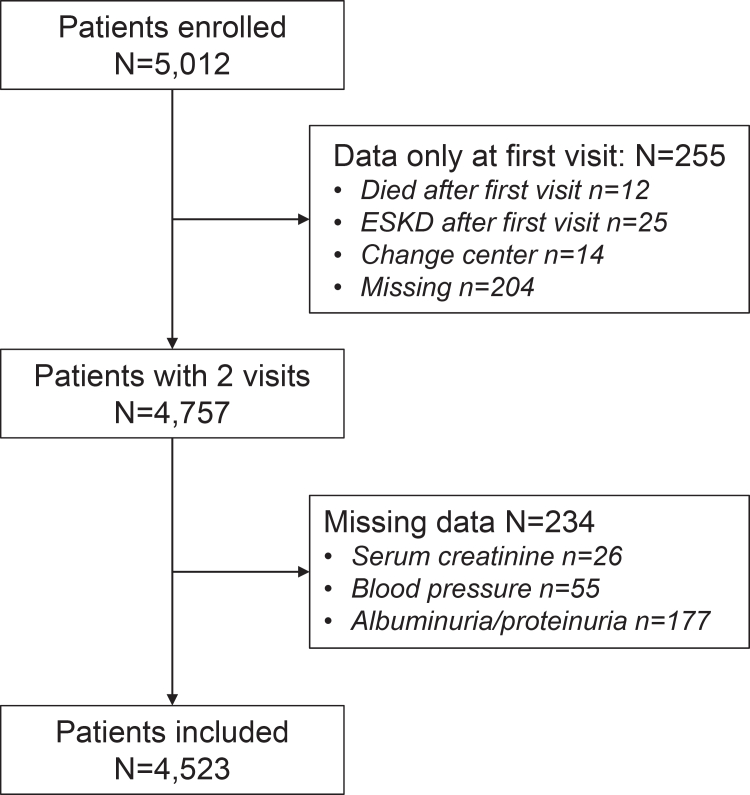
Flowchart of patients. One patient may have more than 1 missing data point. ESKD, end-stage kidney disease.

Out of the initial cohort of 5012 consecutive patients with ND-CKD, 4523 patients were included in the analysis (Figure 1). Table 1 describes the baseline characteristics of patients, which were collected a median of 18.6 months after the first visit to the clinic. Male sex was predominant, and patients were generally older, with 43.4% patients aged > 75 years; mean eGFR was 34 ml/min per 1.73 m^2^, and median ACR was 70 mg/g. Overall, hypertension, glomerulonephritis, and diabetic nephropathy were similarly prevalent causes of CKD.

**Table 1:** Demographic and basal clinical characteristics in the whole cohort and by diabetic status

Parameters	Overall (*n* = 4523)	No diabetes (*n* = 2716)	Diabetes (*n* = 1807)	*P*
Age (yrs)	70.6 ± 13.9	68.2 ± 15.5	74.1 ± 10.1	< 0.001
Age > 75 yrs (%)	43.4	39.3	49.6	< 0.001
Male sex (%)	65.4	63.3	68.6	< 0.001
Previous nephrology care (mos)	18.6 (6.0–54.8)	18.4 (6.0–57.3)	19.1 (6.0–51.3)	0.379
BMI (kg/m^2^)^a^	26.0 ± 4.9	25.3 ± 4.6	27.1 ± 5.1	< 0.001
BMI > 30 kg/m^2^ (%)	18.0	13.2	25.3	< 0.001
Active smokers (%)^b^	11.1	11.1	11.2	0.873
Educational level (%)				< 0.001
Primary	24.0	21.3	28.2	
Lower secondary	32.8	31.3	35.1	
Upper secondary	32.4	37.4	28.8	
University	10.8	12.7	7.8	
Diabetes (%)	40.0	-	40.0	-
Type 1	2.2	-	2.2	
Type 2	97.8	-	97.8	
History of CVD (%)	39.9	32.2	51.6	< 0.001
Coronary artery disease	19.4	13.8	27.8	
Peripheral artery disease	14.3	10.9	19.5	
Atrial fibrillation	14.2	12.4	16.9	
Congestive heart failure	9.7	8.0	12.2	
Cerebrovascular disease	7.2	6.3	8.5	
Cause of renal disease (%)				< 0.001
Hypertension	13.4	16.3	9.0	
Diabetic nephropathy	11.2	-	28.1	
Glomerulonephritis	12.2	17.5	4.3	
Polycystic kidney disease	3.0	4.7	0.4	
Tubulo-interstitial nephritis	3.4	4.6	1.5	
Other	21.0	24.2	16.1	
Unknown	35.8	32.6	40.7	
eGFR (ml/min per 1.73 m^2^)	33.7 ± 18.6	34.4 ± 20.0	32.6 ± 16.2	< 0.001
ACR (mg/g)	70 (11–350)	65 (9–339)	80 (15–394)	0.002

ACR, urinary albumin creatinine ratio; BMI, body mass index; CVD, cardiovascular disease; eGFR, estimated glomerular filtration rate; IQR, interquartile range.

Data are mean ± SD, median (IQR), or percentage.

a Available in 4490 patients (2701 without diabetes and 1789 with diabetes).

b Available in 4488 patients (2704 without diabetes and 1784 with diabetes).

**Figure 2:**
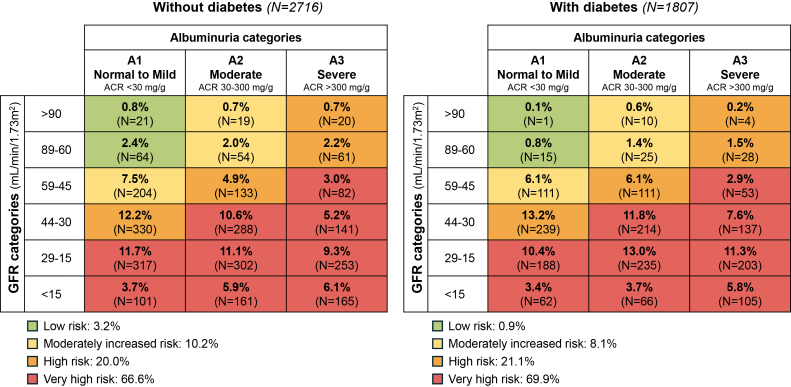
Risk profile according to the Kidney Disease: Improving Global Outcomes 2024 in patients with diabetic and nondiabetic chronic kidney disease. ACR, urinary albumin-to-creatinine ratio; GFR, glomerular filtration rate.

The cohort was characterized by a high prevalence of severe CKD. Specifically, patients were mostly classified as CKD stage 3b to 5 (stage 1–2, 7.1%; stage 3a, 15.3%; stage 3b, 29.8%; stage 4, 33.1%; and stage 5, 14.6%). The anticipated cardiorenal risk was high or very high in majority of patients, that is, 86.6% of nondiabetic and 91.0% of patients with diabetes (Figure 2). The severity of disease was supported by the main characteristics of patients; advanced age, male gender, low educational level, high body mass index, and a history of cardiovascular disease were common in this cohort, with higher prevalence of diabetic individuals that represented 40% of the entire population (Table 1). As expected, cardiorenal risk increased with age; specifically, the age-driven increase of risk was mainly related to lower eGFR, whereas the contribution of ACR became more evident with CKD stage 3b to 5 (Supplementary Figure S1).

**Figure 3:**
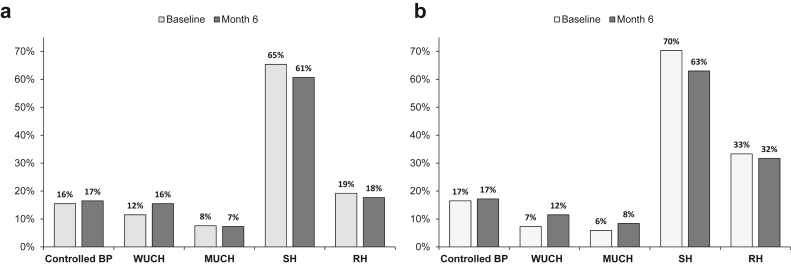
Prevalence of BP profiles and RH in patients without (panel A) and with (panel B) diabetes with available home BP measurements.∗ ∗Available in 1586 patients, 962 without diabetes and 624 with diabetes. BP, blood pressure; MUCH, masked uncontrolled hypertension; RH, resistant hypertension; SH, sustained hypertension; WUCH, white-coat uncontrolled hypertension.

Analysis of BP control showed that only 1 of 3 patients reached the goal at either visit (Table 2). Home BP, available in 1586 patients, revealed that sustained hypertension was the most prevalent pattern of BP profile (> 60% patients at the 2 visits) and a significant prevalence of RH, especially in patients with DM (∼ 80% higher vs. the nondiabetic subgroup) (Figure 3). Office BP < 140/90 mm Hg was detected at baseline in 60.9% of patients without DM and 57.1% of those with DM, with an increase at month-6 visit to 64.6% and 61.4%, respectively.

**Table 2:** Blood pressure and related treatment in patients stratified by diabetic status in the 2 visits

Parameters	No diabetes (*n* = 2716)		Diabetes (*n* = 1807)	
	Baseline	Month 6	Baseline	Month 6
Systolic OBP (mm Hg)	132 ± 17	130 ± 16^a^	134 ± 18	132 ± 17^a^
Diastolic OBP (mm Hg)	76 ± 11	75 ± 10^a^	75 ± 10	74 ± 10^a^
OBP < 130/80 mm Hg (%)	28.5	31.2^a^	26.1	29.6^a^
Systolic HBP (mm Hg)^b^	127 ± 11	125 ± 11^a^	128 ± 11	126 ± 11^a^
Diastolic HBP (mm Hg)^b^	75 ± 8	75 ± 8^a^	74 ± 8	74 ± 8
HBP < 130/80 mm Hg (%)	27.0	32.0^a^	23.8	28.6^a^
UNa (mEq/24 h)^c^	151 ± 66	142 ± 53^a^	154 ± 68	139 ± 62^a^
UNa ≤ 100 mEq/24 h (%)	27.0	30.5	27.6	34.4
Number of antihypertensive drugs	2.17 ± 1.20	2.15 ± 1.22	2.65 ± 1.19	2.62 ± 1.22
0 (%)	7.1	7.6	3.6	4.5
1 (%)	23.4	23.9	12.7	13.2
2 (%)	31.4	30.7	28.0	27.3
3 (%)	25.6	25.0	32.6	31.4
≥ 4 (%)	12.5	12.7	23.1	23.6
Class of antihypertensive drugs (%)				
RAS inhibitors	62.9	62.3	66.8	65.7
Loop diuretics	34.1	35.2^a^	50.5	51.6
Calcium channel blockers	43.6	43.3	50.6	51.2
Beta-blockers	43.3	42.9	58.6	58.3
Thiazide diuretics	8.2	7.6	10.8	9.5^a^
Other	24.9	23.7	27.7	25.7

HBP, home blood pressure; IQR, interquartile range; OBP, office blood pressure; RAS renin-angiotensin system; UNa, urinary sodium excretion.

Data are mean ± SD, median (IQR), or percentage.

a *P* < 0.05 vs. baseline.

b Available in 1,586 patients (962 without diabetes and 624 with diabetes).

c Available in 945 patients (575 without diabetes and 370 with diabetes).

Analysis of antihypertensive therapy showed that at both visits, majority of patients were treated with ≥ 2 drugs (Table 2). The most prevalent agents were RAS inhibitors, prescribed in > 60% patients at each visit, with a slight therapy intensification at month-6 visit. About 1 of 3 patients was adherent to a low-salt diet, as testified by 24-hour urine collection available for 945 patients.

As depicted in Table 3, albuminuria significantly decreased between the 2 visits in all patients, with an improved distribution of ACR categories in those without DM (12.9%, *P* = 0.001) and with DM (15.4%, *P* = 0.002). However, severe ACR (> 300 mg/g) was still detected in 26% and 24% patients who were diabetic and nondiabetic, respectively. In addition to improved BP control, increased use of SGLT2i in the 2 subgroups of patients and GLP1-RAs in the diabetic subgroup was observed in parallel with the reduction of ACR. Only a small minority of patients with DM were treated with finerenone.

**Table 3:** Main laboratory parameters in patients stratified by diabetic status in the 2 visits

Parameters	No diabetes (*n* = 2716)		Diabetes (*n* = 1807)	
	Baseline	Month 6	Baseline	Month 6
eGFR (ml/min per 1.73 m^2^)	34.4 ± 20.0	34.1 ± 20.3^a^	32.6 ± 16.2	32.3 ± 16.4
HbA1c (*%*)	-		6.92 ± 0.97	6.72 ± 0.99^a^
HbA1c < 7% (%)			60.2	70.5^a^
ACR (mg/g)	65 (9–339)	61 (10–300)^a^	80 (15–394)	68 (14–337)^a^
ACR categories (%)				
A1	38.2	39.0	34.1	35.6
A2	35.2	36.7	36.6	38.0
A3	26.6	24.2	29.3	26.4
Antialbuminuric drugs (%)				
RAS inhibitors	62.9	62.3	66.8	65.7
SGLT2-inhibitors	15.3	18.9^a^	39.7	43.7^a^
GLP1-RA	0.3	0.3	20.2	21.4^a^
Steroidal MRA	5.1	4.9	7.9	7.8
Finerenone	-	-	0.9	2.5^a^

GLP1-RA, glucagon-like peptide-1 receptor agonist; HbA1c, glycated hemoglobin; IQR, interquartile range; MRA, mineralocorticoid receptor antagonist; RAS renin-angiotensin system; SGLT2, sodium-glucose cotransporter 2.

Data are mean ± SD, median (IQR), or percentage.

a *P* < 0.05 versus baseline.

Table 4 shows the areas of therapeutic improvement related to antialbuminuric agents, quantified based on the lack of intensification of prescribed therapy at month 6 despite guideline indications. A remarkable therapeutic inertia was detected for the 4 classes of nephroprotective agents. When examining the factors associated with therapeutic inertia in the prescription of the 2 main classes of drugs indicated in patients with and without DM, we found that lower BP and eGFR < 30 ml/min per 1.73 m^2^ were associated with greater inertia for RAS inhibitors; whereas eGFR < 45 ml/min per 1.73 m^2^, negative history of DM and cardiovascular disease, low ACR, and older age associated with greater inertia in the prescription of SGLT2i (Supplementary Table S1).

**Table 4:** Areas of improvement in the use of nephroprotective drugs in patients stratified by diabetic status^a^

Groups	RAS inhibitors	SGLT2i	GLP1-RA	Finerenone
Patients without diabetes	2716	2716	2716	2716
Untreated with drug at baseline	1006	2302	-	-
Eligible patients	284	1,456	-	-
Drug added at month 6	41	118	-	-
Prevalence of therapeutic inertia at month 6	85.6%	91.9%	-	-
Patients with diabetes^b^	1768	1768	1768	1768
Untreated with drug at baseline	586	1,056	1,406	1,752
Eligible patients	202	687	365	530
Drug added at month 6	33	106	24	20
Prevalence of therapeutic inertia at month 6	83.7%	84.6%	93.4%	96.2%

GLP1-RA, glucagon-like peptide-1 receptor agonist; RAS renin-angiotensin system; SGLT2, sodium-glucose cotransporter 2.

a See text for methodological details. The prevalence of therapeutic inertia for each drug class was calculated as (1 – [drug added at month 6/eligible patients] × 100).

b Only patients with type 2 diabetes mellitus were considered.

## Discussion

This study originally provides data on the largest real-world cohort of patients with ND-CKD under regular nephrology care enrolled after the publication of the current guidelines on ND-CKD management.^7^, ^8^, ^9^, ^10^, ^11^^,^^24^ The population examined was characterized by a severe cardiorenal prognosis, particularly in those aged > 65 years and lower eGFR (Supplementary Table S1). Of note, the observed risk profile has worsened compared with studies conducted 15 to 20 years ago that had similar selection criteria and procedures (Supplementary Table S2).^29^ We observed a major increase in age and in the prevalence of DM. Conversely, the prevalence of moderate-to-severe ACR decreased, whereas the normoalbuminuric category increased; because this occurred in the presence of similar eGFR and a reduction of BP levels and RAS inhibitor use, it is possible to hypothesize that a higher frequency of atherosclerotic kidney damage correlated with population aging.^30^ These findings are supported by a recent meta-analysis from our group that provided, as ancillary data, information on the change of risk profile of the standard-of-care arm of 92 randomized controlled trials in ND-CKD published over the past 30 years by disclosing a progressive increase in mean age and DM prevalence, together with progressively lower baseline mean systolic BP and 24-hour proteinuria.^31^

The high-risk profile, predominantly related to unmodifiable determinants of prognosis, was further worsened by the high prevalence of patients out-of-target for the 2 major modifiable determinants of kidney outcome, that is, hypertension and ACR. Specifically, office BP out-of-target was detected at month-6 visit in > 70% of the cohort (Table 2), with sustained hypertension and RH being the 2 prevalent components of the BP profile evaluated by means of available home measurements in 35% of cohort (Figure 3). The findings of home BP measurements are critical to identify areas of improvement. In ND-CKD under nephrology care, home BP acts as stronger predictor of end-stage kidney disease or death than office BP.^32^ Moreover, out-of-office BP above target despite office BP being at target predicts a higher cardiorenal risk similar to that observed when both BP measurements are above target.^33^ Although trials aimed at guiding therapy intensification based on home BP have not yet been published, recent evidence has demonstrated the feasibility and safety of intensive home BP lowering in advanced CKD.^34^ Of even greater importance is the observed high prevalence of RH, which at month 6 was a remarkable feature of patients without (18%) and especially in those with DM (32%). We previously demonstrated, in a similar cohort of 436 patients with ND-CKD followed by nephrologists, a prevalence of RH of 23%, which was associated with a 2-fold higher risk of cardiovascular events and an almost 3-fold higher risk of KRT compared with controls.^35^

Similar to BP control, we found that > 60% of patients had high ACR (Table 3). A slight but significant decrease in ACR was detected at month 6 versus baseline; however, > 1 of 4 patients was left with severe albuminuria at the second visit. This finding is remarkable because the nephrology community is well aware for almost 15 years of the independent predictive role of albuminuria, even in the lower range, on kidney and cardiovascular outcomes.^36^, ^37^, ^38^ The results of these historical analyses have now been supported by evidence from several randomized controlled trials showing that approximately 70% of renal risk reduction in the long-term is explained by the antialbuminuric response in the initial 6 months; and that a 25% decrease of albuminuria, which is an antialbuminuric effect definitely larger than that observed in the present study, is the target to reach to improve kidney survival.^39^

The evaluation of the therapeutic approach allowed us to identify the potential areas of improvement in the control of hypertension and albuminuria. At month 6, only 38% of patients without DM and 55% with DM were treated with ≥ 3 BP-lowering drugs, with only 43% and 61%, respectively, under diuretic therapy though the adherence to a low-salt diet was limited to only 1 of 3 patients (Table 2). These data contrast with recommendations of guidelines,^9^, ^10^, ^11^^,^^24^ as well as with the knowledge that extracellular volume expansion is the main cause of CKD-related hypertension.^40^ Suboptimal BP control in office, coupled with significant aging of the population, may suggest that nephrologists do not intensify therapy because of concerns for normotensive ischemic acute kidney failure that can occur in elderly patients with CKD, especially if under RAS inhibitor therapy.^41^^,^^42^

Greater attention should be dedicated to antialbuminuric therapy, not only for the strong and consistent association of albuminuria with cardiorenal outcome, but also because in renal clinics the prognostic value of residual albuminuria definitely overcomes that of suboptimal BP control.^43^ Current guidelines identify RAS inhibitors and SGLT2i as the 2 main classes of antialbuminuric (and nephroprotective) agents.^7^, ^8^, ^9^, ^10^, ^11^ At the time of data collection, the Italian Health System offered full coverage of these agents in patients with albuminuric CKD regardless of diabetes status and of the second-line agents, GLP1-RA and finerenone, in diabetes only. Notwithstanding, therapeutic inertia for the RAS inhibitors and SGLT2i, respectively detected in 85% and 90% of eligible patients, clearly emerged as a major issue. In contrast, the larger inertia for GLP1-RAs and finerenone was likely dependent on the approval of their reimbursement few months before the study start (Table 4).

In particular, prescription of RAS inhibitors remained unchanged in the 2 visits with 62% of nondiabetic and 66% of patients with diabetes being treated at month 6 (Table 3). The adherence to guidelines in our cohort is better than that observed in USA, where RAS inhibitors are prescribed to less than half of pateints with albuminuria.^44^ Interestingly, in the present study, we observed a lower use of RAS inhibitors than in the past (Supplementary Table S2). Again, it is possible that concerns for normotensive ischemic acute kidney failure in this elderly cohort may have played a role; BP in the low-normal range, in fact, was associated with 3-fold higher odds of therapeutic inertia for RAS inhibitors (Supplementary Table S1). Similarly, eGFR < 30 ml/min per 1.73 m^2^ was associated with more than double odds of therapeutic inertia; this finding is in contrast with the trial-level evidence that this class of drugs increases kidney survival in advanced disease.^45^

In our cohort, though the prescription of SGLT2i increased across the 2 study visits, > 80% of patients without DM and 55% of those with DM were left untreated (Table 3), with frequent occurrence of therapeutic inertia at the month-6 visit (Table 4). The difference between DM and non-DM may be ascribed to the earlier availability of SGLT2i in patients with DM. As expected, inertia was greater in individuals without DM or cardiovascular disease, and in the presence of low-normal ACR (Supplementary Table S1). More importantly, multivariate analysis showed that nephrologists are less prone to prescribe SGLT2i in patients with older age or eGFR < 45 ml/min per 1.73 m^2^. Specifically, age > 75 years was associated with a 70% higher risk of therapeutic inertia. Although these agents slow CKD progression and improve cardiovascular outcomes in older as in younger patients with a similar safety profile, the issue is still debated.^46^ More complex to interpret is the observation that these drugs are less likely to be prescribed starting from eGFR < 45 ml/min per 1.73 m^2^, that is, a level 25 ml/min higher than the lowest recommended threshold for therapy start.^8^, ^9^, ^10^, ^11^ This holds particularly true when considering that large meta-analyses demonstrated that SGLT2i are still effective and safe in patients with eGFR < 30 ml/min per 1.73 m^2^.^47^^,^^48^

A limitation of this study, which was meant to represent contemporary nephrology practice, is that we evaluated prescription rather than adherence to therapy; nevertheless, this potential bias becomes irrelevant when considering that the prescribed therapy was *per se* inadequate. In addition, the presence in Italy of a universal coverage for drug dispensation may reduce the generalizability of our findings to those countries with different health care systems. Finally, our findings may not apply to patients not followed-up with by nephrologists. The strengths of the study are the enrollment of a large contemporary cohort of patients with ND-CKD under stable nephrology care, the collection of data in the renal clinics obtained in 2 visits with 6-month interval, and the universal health coverage in Italy as well.

In conclusion, this study provides evidence that patients under nephrology care are today characterized by a high or very high cardiorenal risk profile, that, moreover, has significantly worsened in the last 2 decades. Despite the worrisome risk profile and the evidence of the benefits of traditional and innovative nephroprotective drugs, most patients remain undertreated because of a pervasive therapeutic inertia that results in a high prevalence of uncontrolled hypertension and abnormal albuminuria. In particular, out-of-office BP appears to be a major area for improvement in terms of monitoring and control to abate sustained, and especially RH. Similarly, it is urgent to reduce the gap between guideline-directed antialbuminuric therapy and nephrology clinical practice. In this regard, real-world studies on efficacy and safety of nephroprotective drugs appear to be as important as randomized controlled trials to identify potential emerging issues when translating trial evidence into daily practice; or alternatively, to make clinical nephrologists more confident with guidelines’ recommendations.

## Appendix

### List of RENEW-CKD investigators

Loreto Gesualdo, Pietro Cirillo, Alessandro Mascolo, Silvia Porreca, Giuseppe Scarimbolo, Davide Morisco, Simone Di Pace (Bari); Gaetano La Manna, Irene Capelli, Miriam Di Nunzio (Bologna); Federico Alberici, Federica Mescia, Francesco Ravelli, Martina Tedesco, Marco Gregori (Brescia); Luigi Vernaglione, Flavia Capaccio, Maria Teresa Farina, Domenica Biasi, Giuseppe Leonardi, Mariagrazia Arcidiacono, Lucia Argentiero, Alessandra Spinelli (Brindisi); Gianfranca Cabiddu, Stefania Maxia, Stefania Caira (Cagliari); Marcellino Corvinelli, Lucio Polese (Campobasso); Vincenzo Bellizzi, Caterina Saviano, Ida Molfino, Pasqualina Acconcia (Caserta); Carmelita Marcantoni, Simone Di Lorenzo, Irene Torrisi, Roberta Aliotta, Grazia Portale (Catania); Mario Bonomini, Bianca Lo Giudice, Emanuele Marini, Lorenzo Nolletti, Valeria Vezzani, Vittorio Sirolli (Chieti); Michele Provenzano, Chiara Summaria, Fiorella Iorio, Francesco Zingone, Giuseppe Pezzi, Roberta Arena (Cosenza); Giovanni Stallone, Dario Troise Barbara Infante (Foggia); Maura Ravera (Genova); Domenico Santoro, Chiara Casuscelli, Michela Calderone, Felicia Cuzzola (Messina); Maurizio Gallieni, Luca Della Volpe, Michela Tedesco, Alessia Guarino, Laura Cosmai, Marco Heidempergher, Sabrina Caruso (ASST Fatebenefratelli Sacco, Milan); Mario Cozzolino, Sara Masotto, Anna Maria Ferrigno, Clorinda Rossi, Chiara Cuffaro, (ASST Santi Paolo e Carlo, Milan); Giuseppe Castellano, Simone Vettoretti (Ospedale Maggiore Policlinico, Milan); Luca De Nicola, Roberto Minutolo, Paolo Tino Ambrosino, Simona Andriella, Davide Cesarano, Annarita D’Ambra, Federica Marzano, Christian Nardelli, Chiara Ruotolo (Department of Advanced Medical and Surgical Sciences, University of Campania, Naples); Antonio Pisani, Fabio Esposito, Michela Saggese, Giuditta della Corte (University Federico II, Naples); Mariadelina Simeoni, Davide Loffredo, Angelo Savino (Department of Translational Medical Sciences, University of Campania, Naples); Felice Nappi, Carla Clienti, Antonietta La Verde (Nola); Angelo Ferrantelli (Palermo); Ciro Esposito, Marta Arazzi, Vittoria Esposito, Filippo Sangregorio (Pavia); Gianpaolo Reboldi, Sara Battistoni, Nicola Lommano, Manuel Burdese (Perugia); Yuri Battaglia, Federica Baciga, Michela Erlati, Veronica Brunelli (Peschiera del Garda); Adamasco Cupisti, Domenico Giannese, Claudia D’Alessandro, Vincenzo Panichi (Pisa); Alessandro Capitanini, Adriano Carmelo Piluso, Dritan Curi, Elana Romoli, Vincenzo Miniello, Brunilda Xhaferi (Pistoia); Giuseppe Grandaliano, Viola D’Ambrosio (Roma); Filippo Aucella, Rachele Grifa, Maria Nardella, Pérez Ys Aurora Del Mar (S. Giovanni Rotondo); Cataldo Abaterusso, Michelangelo Beltrami, Marta Guizzo, Roberta Lazzarin, Mariapaola Protti, Claudia Ungaro (Treviso); Pietro Manuel Ferraro, Isabella Squarzoni, Andrea Spasiano, and Francesco Bonetti (Verona).

## Disclosure

LDN has received fees from AstraZeneca for lectures, consulting fees from Bayer, and support for attending meetings from Amgen. GLM has received fees as advisory board member for Eli-Lily and is an invited speaker at meetings supported by Astellas, Hansa Biopharma, Travere, CSL Vifor, Eli-Lily, and GlaxoSmithKline. VB served as a consultant, advisory board member, and invited speaker for Dr Shar and Fresenius Kabi and as an invited speaker for AstraZeneca. LG has been member of advisory boards for Amgen, AstraZeneca, GlaxoSmithKline, Novartis, Chinook, Roche, Bayer, CSL Vifor, and Boehringer and an invited speaker at meetings supported by Bayer, Astra Zeneca, and Kabi-Fresenius. FN has served as invited speaker for Amgen and AstraZeneca. PMF received consultant fees and grant and other support from Allena Pharmaceuticals, Alnylam, Amgen, AstraZeneca, Bayer, Boehringer, CSL Vifor, Gilead, EG Stada, Novartis, Novo Nordisk, Otsuka Pharmaceuticals, Rocchetta, and Vifor Fresenius and received royalties as an author for UpToDate. CM has been member for advisory board for Novartis and CSL Vifor, and invited speaker at meetings supported by AstraZeneca. DS has been member of advisory boards for CSL Vifor, Bayer, Sobi, Amgen, Novartis, and Boheringer and is an invited speaker at meetings supported by Otsuka, GlaxoSmithKline, Alexion, and AstraZeneca. AP has received fees for lectures and symposia, participation on advisory boards and consultancy from AstraZeneca, Sanofi, and Farmanutra. ACu has received consultancy fees from Dr. Shar and Fresenius Kabi. MR has been member of advisory boards for Amgen and Boehringer and is an invited speaker at meetings supported by Amgen, Astellas, AstraZeneca, Boehringer, and CSL Vifor. MC has been a member of advisory boards for Amgen, Novo Nordisk, CSL Vifor, and Boehringer and is an invited speaker at meetings supported by Amgen, Astellas, Astra Zeneca, Bayer, Boehringer, and CSL Vifor. MG has been member of advisory boards for CSL Vifor, BD, and Sanofi and an invited speaker at meetings supported by CSL Vifor, Vantive, Amicus, and Sanofi. FAl has received fees from CSL Vifor for lectures; consulting fees from Alexion, Novartis, CSL, and Amicus Therapeutics; and research grant from Sanofi. YB has received fees for lectures from AstraZeneca and Astellas. ACa has served as invited speaker for AstraZeneca. FAu has received fees from AstraZeneca for lectures and consulting fees from Amgen. GC has been invited speaker at meetings supported by Estor, Fresenius, and Novartis. MS has been invited speaker at meetings supported by AstraZeneca and Boehringer. MP has been invited speaker at meetings supported by AstraZeneca, Bayer, and Menarini. MB has been member of advisory boards for Amgen, Astellas, AstraZeneca, and GlaxoSmithKline and has received fees from Amgen and Astellas for lectures. GR has served as invited speaker for Novartis. CE has been a member of advisory boards for Amgen, Astellas, Bayer, and AstraZeneca; an invited speaker at meetings supported by Amgen, Astellas, AstraZeneca, Bayer, and CSL Vifor; and support for attending meetings from CSL Vifor. GG has been member of advisory boards for AstraZeneca, Bayer, and Boehringer; RM has been member of advisory boards for Amgen, Astellas, and Bayer, Boehringer; an invited speaker at meetings supported by Amgen, Astellas, AstraZeneca, Bayer, Boehringer, Novo Nordisk, and CSL Vifor; and support for attending meetings from Amgen. All the other authors declared no competing interests.

## Supplementary Material

Supplementary File (PDF) **Figure S1.** Distribution of eGFR and ACR categories according to age strata. **Table S1.** Factors associated with therapeutic inertia in the prescription of RAS inhibitors and SGLT2 inhibitors. **Table S2.** Change over time in the main features of patients with ND-CKD with stage 3 to 5 disease managed in Italian renal clinics. STROBE checklist.

## Footnotes

Supplementary File (PDF)
**Figure S1.** Distribution of eGFR and ACR categories according to age strata. **Table S1.** Factors associated with therapeutic inertia in the prescription of RAS inhibitors and SGLT2 inhibitors. **Table S2.** Change over time in the main features of patients with ND-CKD with stage 3 to 5 disease managed in Italian renal clinics. STROBE checklist.
