# Supplementary material for: Missing Opportunity for Nephroprotective Therapy in Patients With Non-Dialysis CKD Under Stable Nephrology Care
Source: Kidney Int Rep. 2026 Apr 14;11(6):106541. doi: 10.1016/j.ekir.2026.106541 (PMC13194180; doi:10.1016/j.ekir.2026.106541)
Supplement: Supplementary File (PDF) — Figure S1. Distribution of eGFR and ACR categories according to age strata. Table S1. Factors associated with therapeutic inertia in the prescription of RAS inhibitors and SGLT2 inhibitors. Table S2. Change over time in the main features of patients with ND-CKD with stage 3 to 5 disease managed in Italian renal clinics. STROBE checklist. [file mmc1.pdf]

Supplementary material for the manuscript

Missing opportunity for nephroprotective therapy in non-dialysis CKD patients under stable nephrology care

By Luca De Nicola et al

Supplementary Figure S1: Distribution of eGFR and ACR categories according to age strata

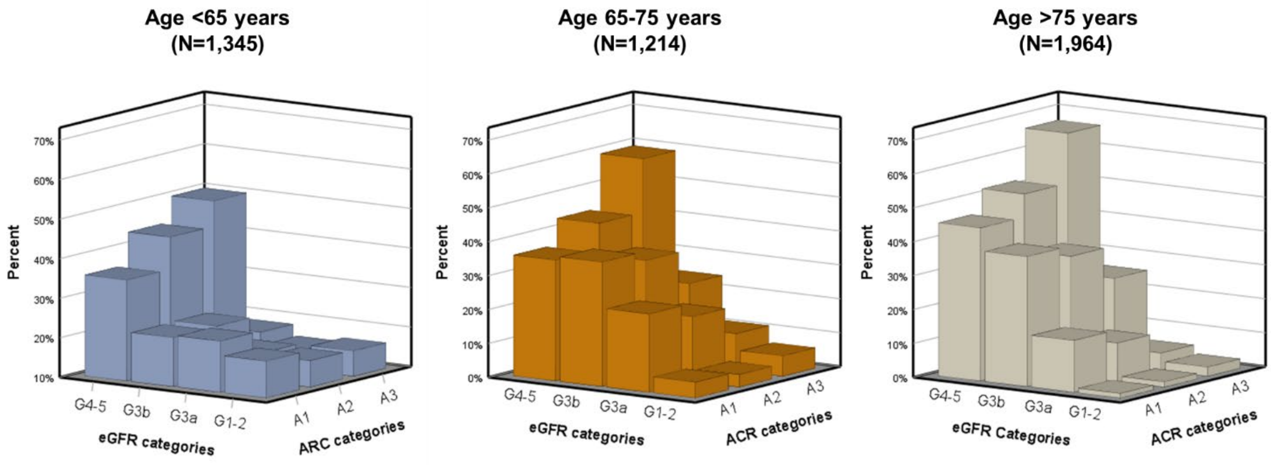

Supplementary Table S1: Factors associated with therapeutic inertia to the prescription of RAS inhibitors and SGLT2 inhibitors

	**Therapeutic inertia for RAS inhibitors**			**Therapeutic inertia for SGLT2 inhibitors**		
	OR	95%CI	P	OR	95%CI	P
Age >75 years (yes vs no)	1.15	0.65-2.05	0.625	**1.70**	**1.24-2.34**	**0.001**
Male sex	1.26	0.71-2.22	0.427	0.81	0.59-1.11	0.192
Body Mass Index (kg/m^2)	0.99	0.94-1.05	0.843	0.98	0.95-1.01	0.226
Systolic BP <130 mmHg (yes vs no)	**3.51**	**1.88-6.55**	**<0.001**	1.20	0.89-1.63	0.240
Type 2 Diabetes (yes vs no)	1.00	0.58-1.73	0.998	**0.48**	**0.36-0.64**	**<0.001**
Prior cardiovascular disease (yes vs no)	0.74	0.55-1.01	0.056	**0.81**	**0.67-0.98**	**0.027**
ACR (g/g)	0.86	0.68-1.09	0.213	**0.75**	**0.65-0.87**	**<0.001**
eGFR>60 mL/min/1.73m^2	Ref.			Ref		
eGFR 45-59 mL/min/1.73m^2	1.86	0.66-5.28	0.234	1.67	0.93-2.99	0.086
eGFR 30-44 mL/min/1.73m^2	1.73	0.70-4.27	0.234	**1.76**	**1.01-3.10**	**0.048**
eGFR 20-29 mL/min/1.73m^2	**2.86**	**1.12-7.29**	**0.028**	**2.56**	**1.42-4.61**	**0.002**

Data are odds ratio (OR) and 95% confidence intervals (95%CI). BP, blood pressure; ACR, urinary albumin creatinine ratio; eGFR, estimated glomerular filtration rate

Supplementary Table S2: Change over time of main features of ND-CKD patients stage 3 to 5 managed in Italian renal clinics

	**RENEW-CKD (N=4655)**	**MULTI-COHORT (N=3957)***
**Enrollment (year)**	**2024**	**2003-2010**
Age (years)	72±13	67±14
Age >75 yrs (%)	47	29.1
Male gender (%)	64.7	59.7
BMI (kg/m^2)	26.0±4.9	27.6±4.9
Smokers (%)	10.6	12.4
Diabetes (%)	42.1	29.3
Previous cardiovascular event (%)	37.9	34.4
Systolic BP *(mmHg)*	133±17	140±19
eGFR *(mL/min/1.73m2)*	31±13	32±13
Albuminuria categories		
A1	40.2	27.7
A2	33.7	24.7
A3	26.1	47.9
Antihypertensive drugs (number)	2.4±1.2	2.3±1.2
RAS inhibitors (%)	64.6	72.2

*From ref. 28. Data are mean±SD or percentage.

Supplementary Data S1: STROBE checklist

	Item No.	Recommendation	Page No.	Relevant text from manuscript
**Title and abstract**	1	(*a*) Indicate the study's design with a commonly used term in the title or the abstract	3	In this multicenter prospective study
		(*b*) Provide in the abstract an informative and balanced summary of what was done and what was found	3	
**Introduction**				
Background/rationale	2	Explain the scientific background and rationale for the investigation being reported	4	
Objectives	3	State specific objectives, including any prespecified hypotheses	5	
**Methods**				
Study design	4	Present key elements of study design early in the paper	6	First paragraph of Methods
Setting	5	Describe the setting, locations, and relevant dates, including periods of recruitment, exposure, follow-up, and data collection	6-7	
Participants	6	(*a*) *Cohort study*—Give the eligibility criteria, and the sources and methods of selection of participants. Describe methods of follow-up<br>*Case-control study*—Give the eligibility criteria, and the sources and methods of case ascertainment and control selection. Give the rationale for the choice of cases and controls<br>*Cross-sectional study*—Give the eligibility criteria, and the sources and methods of selection of participants	7	
		(*b*) *Cohort study*—For matched studies, give matching criteria and number of exposed and unexposed<br>*Case-control study*—For matched studies, give matching criteria and the number of controls per case	N/A	
Variables	7	Clearly define all outcomes, exposures, predictors, potential confounders, and effect modifiers. Give diagnostic criteria, if applicable	7-9	
Data sources/ measurement	8*	For each variable of interest, give sources of data and details of methods of assessment (measurement). Describe comparability of assessment methods if there is more than one group	*N/A*	
Bias	9	Describe any efforts to address potential sources of bias	7	
Study size	10	Explain how the study size was arrived at	6	

Continued on next page

Quantitative variables	11	Explain how quantitative variables were handled in the analyses. If applicable, describe which groupings were chosen and why	9	Patients were grouped by diabetic status being this the main non-modifiable risk factor in CKD
Statistical methods	12	(*a*) Describe all statistical methods, including those used to control for confounding	9	
		(*b*) Describe any methods used to examine subgroups and interactions	9	
		(*c*) Explain how missing data were addressed	9	
		(*d*) *Cohort study*—If applicable, explain how loss to follow-up was addressed<br>*Case-control study*—If applicable, explain how matching of cases and controls was addressed<br>*Cross-sectional study*—If applicable, describe analytical methods taking account of sampling strategy	N/A	
		(*e*) Describe any sensitivity analyses	N/A	
**Results**				
Participants	13*	(a) Report numbers of individuals at each stage of study—eg numbers potentially eligible, examined for eligibility, confirmed eligible, included in the study, completing follow-up, and analysed	10	Details are provided in Figure 1
		(b) Give reasons for non-participation at each stage	Figure 1	
		(c) Consider use of a flow diagram	Done	Figure 1
Descriptive data	14*	(a) Give characteristics of study participants (eg demographic, clinical, social) and information on exposures and potential confounders	10	Results and Table 1
		(b) Indicate number of participants with missing data for each variable of interest	9	
		(c) *Cohort study*—Summarise follow-up time (eg, average and total amount)	N/A	
Outcome data	15*	*Cohort study*—Report numbers of outcome events or summary measures over time	*N/A*	
		Case-control study—Report numbers in each exposure category, or summary measures of exposure		
		Cross-sectional study—Report numbers of outcome events or summary measures		
Main results	16	(*a*) Give unadjusted estimates and, if applicable, confounder-adjusted estimates and their precision (eg, 95% confidence interval). Make clear which confounders were adjusted for and why they were included	Supplementary Table S1	Multivariable logistic regression analysis (OR and 95%CI) was used to identify factors associated with the presence of therapeutic inertia
		(*b*) Report category boundaries when continuous variables were categorized	8	category boundaries for ARR and eGFR are those recommended by KDIGO
		(*c*) If relevant, consider translating estimates of relative risk into absolute risk for a meaningful time period	N/A	

Continued on next page

Other analyses	17	Report other analyses done—eg analyses of subgroups and interactions, and sensitivity analyses	10-11
**Discussion**			
Key results	18	Summarise key results with reference to study objectives	12
Limitations	19	Discuss limitations of the study, taking into account sources of potential bias or imprecision. Discuss both direction and magnitude of any potential bias	15
Interpretation	20	Give a cautious overall interpretation of results considering objectives, limitations, multiplicity of analyses, results from similar studies, and other relevant evidence	12-15
Generalisability	21	Discuss the generalisability (external validity) of the study results	15
**Other information**			
Funding	22	Give the source of funding and the role of the funders for the present study and, if applicable, for the original study on which the present article is based	17

*Give information separately for cases and controls in case-control studies and, if applicable, for exposed and unexposed groups in cohort and cross-sectional studies.

Note: An Explanation and Elaboration article discusses each checklist item and gives methodological background and published examples of transparent reporting. The STROBE checklist is best used in conjunction with this article (freely available on the Web sites of PLoS Medicine at http://www.plosmedicine.org/, Annals of Internal Medicine at http://www.annals.org/, and Epidemiology at http://www.epidem.com/). Information on the STROBE Initiative is available at www.strobe-statement.org.
